# Molecular classification of follicular thyroid carcinoma based on *TERT* promoter mutations

**DOI:** 10.1038/s41379-021-00907-6

**Published:** 2021-09-08

**Authors:** Hyunju Park, Hyeong Chan Shin, Heera Yang, Jung Heo, Chang-Seok Ki, Hye Seung Kim, Jung-Han Kim, Soo Yeon Hahn, Yun Jae Chung, Sun Wook Kim, Jae Hoon Chung, Young Lyun Oh, Tae Hyuk Kim

**Affiliations:** 1 Department of Medicine, Thyroid Center, Samsung Medical Center, Sungkyunkwan University School of Medicine, Seoul, Korea; 2 Department of Pathology, Keimyung University School of Medicine, Daegu, Korea; 3 Green Cross Genome, Yongin, Korea; 4 Statistics and Data Center, Research Institute for Future Medicine, Samsung Medical Center, Sungkyunkwan University School of Medicine, Seoul, Korea; 5 Division of Breast and Endocrine Surgery, Department of Surgery, Samsung Medical Center, Sungkyunkwan University School of Medicine, Seoul, Korea; 6 Department of Radiology, Samsung Medical Center, Sungkyunkwan University School of Medicine, Seoul, Korea; 7 Department of Internal Medicine, Chung-Ang University Hospital, Chung-Ang University College of Medicine, Seoul, Korea; 8 Department of Pathology and Translational Genomics, Samsung Medical Center, Sungkyunkwan University School of Medicine, Seoul, Korea

**Keywords:** Cancer epigenetics, Thyroid diseases

## Abstract

Follicular thyroid carcinoma (FTC) has different clinicopathological characteristics than papillary thyroid carcinoma. However, there are no independent systems to predict cancer-specific survival (CSS) in FTC. Telomerase reverse transcriptase (*TERT*) promoter mutations are associated with tumor aggressiveness. Thus, it could be a potential prognostic marker. The aim of this study was to refine the CSS risk prediction using *TERT* promoter mutations in combination with the fourth edition of World Health Organization (WHO 2017) morphological classification. We investigated 77 FTC patients between August 1995 and November 2020. Cox regression was used to calculate hazard ratios to derive alternative groups. Disease-free survival (DFS) and CSS predictability were compared using Proportion of variation explained (PVE) and C-index. CSS was significantly different in encapsulated angioinvasive (EA)-FTC patients stratified by *TERT* promoter mutations [wild-type (WT-*TERT*) vs. mutant (M-*TERT*); *P* < 0.001] but not in minimally invasive (MI)-FTC and widely invasive (WI)-FTC patients (*P* = 0.691 and 0.176, respectively). We defined alternative groups as follows: Group 1 (MI-FTC with WT-*TERT* and M-*TERT*; EA-FTC with WT-*TERT*), Group 2 (WI-FTC with WT-*TERT*), and Group 3 (EA-FTC with M-*TERT*; WI-FTC with M-*TERT*). Both PVE (22.44 vs. 9.63, respectively) and C-index (0.831 vs. 0.731, respectively) for CSS were higher in the alternative groups than in the WHO 2017 groups. Likewise, both PVE (27.1 vs. 14.9, respectively) and C-index (0.846 vs. 0.794, respectively) for DFS were also higher in the alternative groups than in the WHO 2017 groups. Alternative group harmonizing of the WHO 2017 classification and *TERT* promoter mutations is effective in predicting CSS in FTC patients, thereby improving DFS predictability.

## Introduction

Papillary thyroid carcinoma (PTC) and follicular thyroid carcinoma (FTC) are both derived from the follicular epithelium of the thyroid gland, and they have the ability to concentrate radioactive iodine^1^. Due to this similarity, both PTC and FTC are usually considered differentiated thyroid carcinomas (DTCs). Since FTC occurrence is less common than that of PTC^2^, staging and treatment strategies for DTC are primarily focused on PTC^3^. However, PTC and FTC have markedly different epidemiological, cytological, pathological, genetic, and clinical behavioral characteristics^4–11^.

The World Health Organization (WHO) classification of tumors serves as an international standard for histopathological diagnosis. In a previous WHO classification (WHO 2004), FTCs were divided into minimally invasive and widely invasive types^12^. However, important modifications to the classification of FTC were made in the revised fourth edition of the WHO classification (WHO 2017)^13^. FTCs are now divided into three categories on the basis of the invasive pattern and angioinvasion: minimally invasive (MI-FTC), encapsulated angioinvasive (EA-FTC), and widely invasive (WI-FTC). After the WHO staging system was revised in 2017, 20.4% of FTC patients were re-classified from MI-FTC to EA-FTC. The predictability of disease-free survival (DFS) has improved as a result of this change but not that of cancer-specific survival (CSS)^14^.

A number of risk stratification and staging systems have been propounded with Lang B. H. et al. reporting that the AJCC/TNM system has the best predictability of CSS in FTC patients^15^. However, these stratification systems were developed mainly for patients with PTC, and there is no specific staging system to predict CSS in FTC patients.

Recent studies have identified telomerase reverse transcriptase (*TERT*) promoter mutations that are closely associated with tumor aggressiveness, early recurrence, and cancer specific deaths in patients with thyroid cancer^16–19^. Though these potential prognostic markers are very promising, none of the current recurrence or mortality risk systems incorporate molecular testing results in thyroid cancer stratification. Recently, molecular marker-based risk stratification of thyroid cancer has been proposed to better predict the clinical outcome of the cancer^20–22^. In this study, we refined risk prediction for thyroid cancer using *TERT* promoter mutations and WHO 2017 morphological classification to enhance CSS and DFS predictions.

## Methods

### Study population

From August 1995 to November 2020, 82 consecutive FTC patients who had undergone initial thyroid surgery at Samsung Medical Center and showed *TERT* promotor mutations, as determined by DNA sequencing, were enrolled. Of the 82 patients, we excluded four patients with follicular variant PTC (FV-PTC) and one patient with PTC. Among 77 patients, 59 were female, and 18 were male. Patients with Hürthle cell thyroid carcinoma and poorly differentiated thyroid carcinoma were not included in this study. This study was approved by the Institutional Review Board of Samsung Medical Center (IRB no. 2021-04-085). Informed consent was waived by the committee as it was a retrospective study.

### Clinicopathological data and outcomes

Operating records and final pathologic reports were reviewed to ascertain tumor categories based on the WHO 2017 classification and the eighth edition of the AJCC/TNM classification (TNM-8). In TNM-8, tumors invading strap muscles, subcutaneous soft tissue, larynx, trachea, esophagus, recurrent laryngeal nerve, and prevertebral fascia, or encasing the carotid artery or mediastinal vessel, are classified as gross extrathyroidal extension (ETE). A pathologist (Y.L.O.) at the Department of Pathology reviewed the pathology slides of patients with multifocality or cervical lymph node metastasis to exclude the possibility of misdiagnoses, such as FV-PTC. The status of vascular invasion was also pathologically confirmed, and all patients were reclassified in accordance with the WHO 2017 criteria into one of the following categories: MI-FTC, EA-FTC, and WI-FTC^23^.

DFS was defined as the time from initial surgery to the date of the first structural recurrence. Structural recurrence was defined as persistent or recurrent disease, determined cytologically or pathologically, and/or the presence of highly suspicious metastatic lesions as observed by imaging. CSS was defined as the time from initial surgery to the time of death due to thyroid cancer. Data of patients who died due to other causes were censored at the time of death.

### Detection of *TERT* promotor mutation

Promotor mutations in *TERT* were identified by semi-nested polymerase chain reaction (PCR) and direct Sanger sequencing of the hot spots (chr5:1,295,228 C > T and chr5:1,295,250 C > T) commonly termed C228T and C250T as previously described^24–26^.

### Statistical analysis

Continuous variables were presented as mean with standard deviation (SD), and categorical variables were presented as numbers and percentages. Patients were stratified as per the WHO 2017 classification and the *TERT* promoter mutation status. Cox regression analysis was used to calculate unadjusted hazard ratios (HRs) to predict the outcome of CSS and DFS, thereby deriving alternative prognostic groupings. Survival curves were plotted using the Kaplan–Meier method, and the log-rank test was used to compare survival significance. To estimate the relative validity of predicting CSS and DFS in each of the WHO 2017 categories and the alternative groups, we calculated the proportions of variation explained (PVEs) using the Cox proportional regression model and Harrell’s C-index^21,27,28^. The PVEs (%) range from 0 to 100 with higher percentages indicating better predictability. The maximum value of the C-index was 1.00, and higher values indicated a more accurate predictive capacity. Statistical analysis was executed using R 4.0.4 (Vienna, Austria; http://www.R-project.org/), and SPSS version 25.0 for Windows (IBM, Chicago, IL, USA).

## Results

### Clinical characteristics

A total of 77 patients were included in this study; 39 patients with MI-FTC, 24 patients with EA-FTC, and 14 patients with WI-FTC. The baseline clinicopathological characteristics according to the WHO 2017 classification are described in Table 1. The presence of gross ETE (*P* for trend = 0.023), presence of distant metastasis (*P* for trend <0.001), status of *TERT* promoter mutations (*P* for trend = 0.033), and AJCC/TNM stage (*P* for trend <0.001) were significantly associated with the aggressiveness of the pathological characteristics in the WHO 2017 classification. Sex, age, and primary tumor size were not significantly different between WHO 2017 groups.

**Table 1:** Clinicopathological characteristics of 77 patients according to the WHO 2017 classification.

	MI-FTC (*n* = 39)	EA-FTC (*n* = 24)	WI-FTC (*n* = 14)	*P* for trend
Sex (*n*, %)				
Female	31 (79.5)	17 (70.8)	11 (78.6)	0.767
Male	8 (20.5)	7 (29.2)	3 (21.4)	
Age, year (mean, SD)	40.1 (13.76)	42.1 (18.7)	48.5 (12.2)	0.172*
Size				
Mean, cm (mean, SD)	3.26 (1.59)	4.08 (1.68)	4.65 (3.37)	0.130*
4 cm or less	28 (71.8)	14 (58.3)	9 (64.3)	0.444
More than 4 cm	11 (28.2)	10 (41.7)	5 (35.7)	
Gross ETE				
Absent	39 (100.0)	23 (95.8)	12 (85.7)	0.023
Present	0 (0.0)	1 (4.2)	2 (14.3)	
Distant metastasis				
Absent	39 (100.0)	22 (91.7)	8 (57.1)	<0.001
Present	0 (0.0)	2 (8.3)	6 (42.9)	
*TERT* promoter mutations				
Wild type	35 (89.7)	19 (79.2)	9 (64.3)	0.033
Mutation	4 (10.3)	5 (20.8)	5 (35.7)	
AJCC/TNM 8th stage				
Stage I	39 (100.0)	18 (75.0)	8 (57.1)	<0.001
Stage II	0 (0.0)	4 (16.7)	5 (35.7)	
Stage III/IV	0 (0.0)	2 (8.3)	1 (7.1)	
Surgical extent				
Total	16 (41.0)	17 (70.8)	13 (92.9)	<0.001
Subtotal or lobectomy	23 (59.0)	7 (29.2)	1 (7.1)	
Cumulative RAI dose				
Less than 100 mCi	24 (61.5)	9 (37.5)	1 (7.1)	<0.001
100 mCi or more	15 (38.5)	15 (62.5)	13 (92.9)	

*MI-FTC* minimally invasive follicular thyroid carcinoma, *EA-FTC* encapsulated angioinvasive follicular thyroid carcinoma, *WI-FTC* widely invasive follicular thyroid carcinoma *SD* standard deviation, *ETE* extrathyroidal extension, *TERT* telomerase reverse transcriptase, *AJCC/TNM* American Joint Committee/tumor-node-metastasis, *RAI* radioactive iodine, *P for trend for continuous variables was analyzed using Jonckheere-Terpstra test.

### Prognostic outcomes according to the WHO 2017 classification and *TERT* promoter mutations

**Fig. 1:**
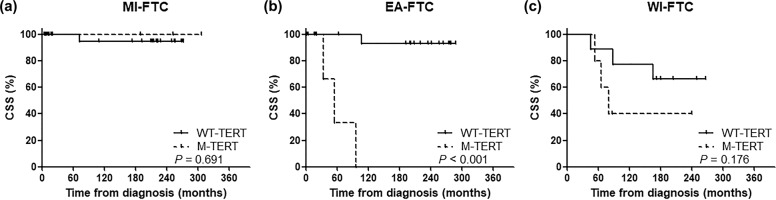
Cancer-specific survival according to the presence of *TERT* promoter mutations in each WHO 2017 group. **a** MI-FTC, **b** EA-FTC, and **c** WI-FTC.

We evaluated CSS in terms of the presence of *TERT* promoter mutations in the three WHO-2017 groups. Most notably, *TERT* promoter mutations were significantly associated with CSS only in the EA-FTC patients (*P* < 0.001) (Fig. 1b). CSS did not differ in the presence of *TERT* promoter mutations in the MI-FTC and WI-FTC patients (Fig. 1a, c). When the patients were stratified according to the WHO 2017 classification system and *TERT* promoter mutational status, the HRs of CSS were found to be higher in the EA-FTC patients with M-*TERT* (HR: 59.09; 95% CI: 5.72–610.68), and the WI-FTC patients with M-*TERT* (HR 23.26; 2.33–231.78), whereas there were no MI-FTC patients with M-*TERT* who died of FTC. In patients with WT-*TERT*, the HRs of CSS increased with increasing pathological aggressiveness as mentioned in the WHO 2017 classification system (Supplementary Table 1).

**Fig. 2:**
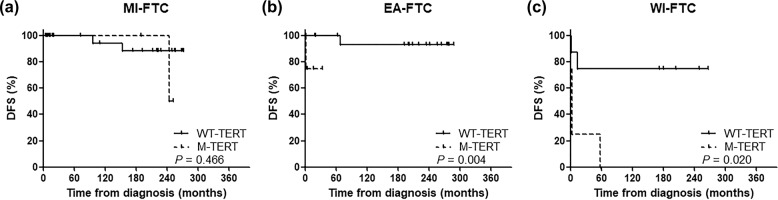
Disease-free survival according to the presence of *TERT* promoter mutations in each WHO 2017 group. **a** MI-FTC, **b** EA-FTC, and **c** WI-FTC.

We also evaluated DFS against the status of *TERT* promoter mutations in the three WHO 2017 groups. In the EA- and WI-FTC patients, DFS was significantly different with differing *TERT* promoter mutational status (*P* = 0.004 and *P* = 0.020, respectively) but not in the MI-FTC patients (*P* = 0.466) (Fig. 2). The pattern of HRs of DFS in the six categories was similar to that of CSS (Supplementary Table 2).

**Fig. 3:**
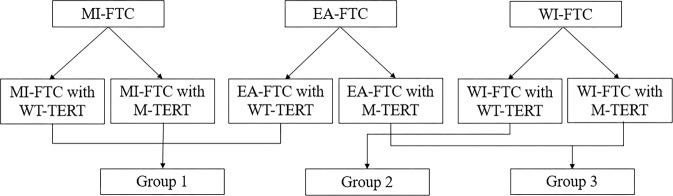
Definitions of the alternative groups. We produced three alternative groups that incorporate the status of *TERT* promoter mutations into the WHO 2017 groups.

After running a comparison among the six categories, we produced the following three alternative groups: Group 1 (MI-FTC with WT-*TERT* and M-*TERT*; EA-FTC with WT-*TERT*), Group 2 (WI-FTC with WT-*TERT*), and Group 3 (EA-FTC with M-*TERT*; WI with M-*TERT*) (Fig. 3). The clinicopathological characteristics between the three alternative groups are shown in Supplementary Table 3. The presence of gross ETE (*P* for trend <0.001), presence of distant metastasis (*P* for trend <0.001), and AJCC/TNM stage (*P* for trend <0.001) were significantly different between the groups.

### Cancer-specific survival according to alternative groups

**Fig. 4:**
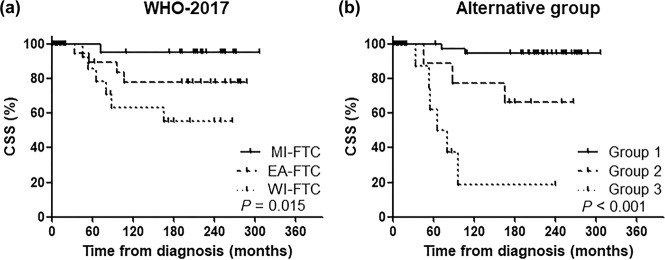
Cancer-specific survival based on the WHO 2017 groups and the alternative groups. **a** the WHO 2017 groups and **b** the alternative groups.

Of the 77 patients, 11 patients died of FTC at a median of 14.8 (interquartile range 1.6–19.7 years) years after the initial operation. Among the WHO 2017 groups, 15-year CSS rates for MI-, EA-, and WI-FTC patients were found to be 95.5, 78.3, and 55.6%, respectively (*P* = 0.015) for which the Kaplan–Meier analysis is shown in Fig. 4a. However, among the alternative groups, the 15-year CSS rates for group 1, 2, and 3 patients were found to be 94.6, 66.7, and 18.8%, respectively (*P* < 0.001) for which the Kaplan–Meier survival curve is shown in Fig. 4b. Table 2 shows the HRs of CSS in the WHO 2017 and alternative groups. Groups 2 (HR 7.09; 95% CI 1.18–42.46) and 3 (HR 32.69; 95% CI 6.25–170.84) showed significantly higher HRs than group 1 (*P* = 0.032 and *P* < 0.001, respectively). The PVEs were 22.44 for the alternative groups and 9.63 for the WHO 2017 groups. The C-index was also higher in the alternative groups than in the WHO 2017 groups (0.831 vs. 0.731, respectively).

**Table 2:** Hazard ratios of cancer-specific survival and predictive capacity according to the WHO 2017 classification versus alternative grouping.

Staging	No of patients (*n*)	No of death (*n*)	CSS 5-year (%)	CSS 10-year (%)	CSS 15-year (%)	Hazard ratio (95% CI)	*P*-value	PVE	C-index
WHO-2017								9.63	0.731
MI-FTC	39	1	100	95.5	95.5	Reference			
EA-FTC	24	4	89.5	78.3	78.3	5.23 (0.58–46.78)	0.139		
WI-FTC	14	6	85.7	63.5	55.6	12.11 (1.45–100.90)	0.021		
Alternative group								22.44	0.831
Group 1	58	2	100	94.6	94.6	Reference			
Group 2	9	3	88.9	77.8	66.7	7.09 (1.18–42.46)	0.032		
Group 3	10	6	62.5	37.5	18.8	32.69 (6.25–170.84)	<0.001		

*WHO* World Health Organization, *MI-FTC* minimally invasive follicular thyroid carcinoma, *EA-FTC* encapsulated angioinvasive follicular thyroid carcinoma, *WI-FTC* widely invasive follicular thyroid carcinoma, *No* number, *CSS* cancer-specific survival, *CI* confidence interval, *PVE* proportion of variation explained.

### Disease-free survival according to alternative groups

**Fig. 5:**
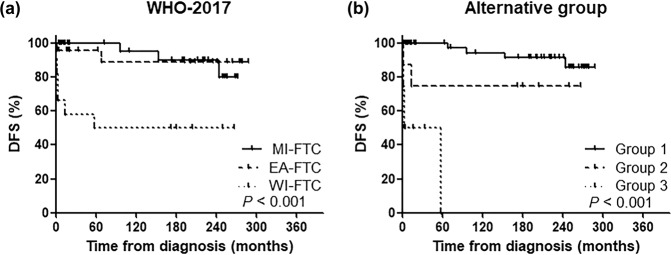
Disease-free survival based on the WHO 2017 groups and the alternative groups. **a** the WHO 2017 groups and **b** the alternative groups.

Figure 5 shows the Kaplan–Meier survival curve for DFS. The 15-year DFS rates for the MI-, EA-, and WI-FTC groups were 90.2, 85.6, and 42.9%, respectively (*P* < 0.001). Among the alternative groups, the 15-year DFS rates for groups 1, 2, and 3 were 91.7, 66.7, and 0.0%, respectively (*P* < 0.001). The HRs of DFS in each of the WHO 2017 and alternative groups are shown in Table 3. Groups 2 and 3 were significantly associated with an increased risk of disease recurrence as compared to group 1 (*P* = 0.037, and *P* < 0.001, respectively). The PVEs in the alternative groups and the WHO 2017 groups were 27.1 and 14.9, respectively. The C-index was also higher in the alternative group than in the WHO 2017 group (0.846 vs. 0.794, respectively).

**Table 3:** Hazard ratios of disease-free survival and predictive capacity according to the WHO 2017 classification versus alternative grouping.

Staging	No of patients (*n*)	No of recur (*n*)	DFS 5-year (%)	DFS 10-year (%)	DFS 15-year (%)	Hazard ratio (95% CI)	*P*-value	PVE	C-index
WHO-2017								14.9	0.794
MI-FTC	39	3	100	95.2	90.2	Reference			
EA-FTC	24	3	91.7	85.6	85.6	1.56 (0.31–7.73)	0.587		
WI-FTC	14	8	42.9	42.9	42.9	9.06 (2.40–34.22)	0.001		
Alternative group								27.1	0.846
Group 1	58	4	100	94.5	91.7	Reference			
Group 2	9	3	66.7	66.7	66.7	4.92 (1.10–22.04)	0.037		
Group 3	10	7	0.0	0.0	0.0	38.80 (8.35–180.27)	<0.001		

*WHO* World Health Organization, *MI-FTC* minimally invasive follicular thyroid carcinoma, *EA-FTC* encapsulated angioinvasive follicular thyroid carcinoma, *WI-FTC* widely invasive follicular thyroid carcinoma, *No* number, *DFS* disease-free survival, *CI* confidential interval, *PVE* proportion of variation explained.

## Discussion

**Fig. 6:**
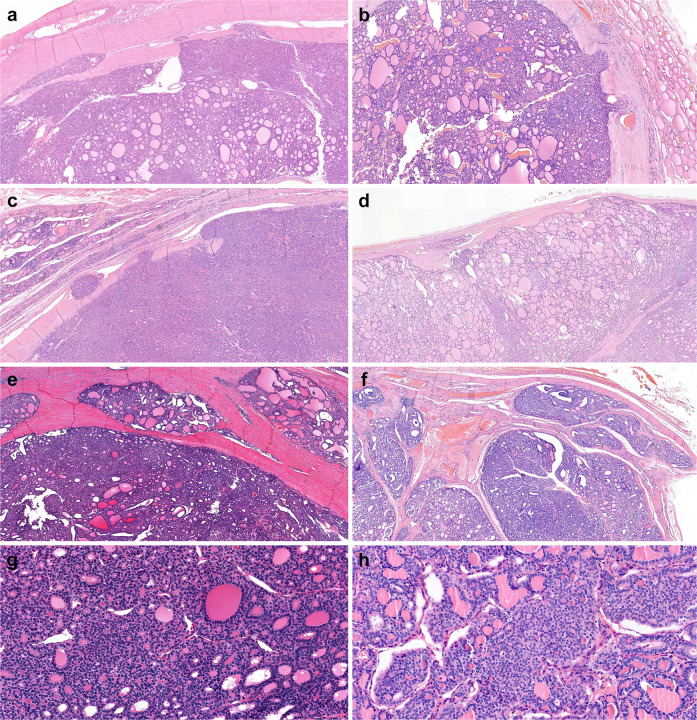
Morphologic features of follicular thyroid carcinoma (FTC). **a** Minimally invasive (MI)-FTC with wild-type *TERT* (WT-*TERT*), **b** MI-FTC with mutant *TERT* (M-*TERT*), **c** encapsulated angioinvasive (EA)-FTC with WT-*TERT*, **d** EA-FTC with M-*TERT*, **e** widely invasive (WI)-FTC with WT-*TERT*, **f** WI-FTC with M-*TERT*, **g** a high magnification of WI-FTC with WT-*TERT*, **h** a high magnification of WT-FTC with *M-TERT* showing focal insular pattern with rare mitotic figures.

The purpose of this study was to assess whether *TERT* promotor mutation can be a new molecular prognostic marker for predicting disease specific survival in FTC patients. We found that the presence of *TERT* promoter mutations was significantly associated with poor survival in the EA-FTC group. Thus, we defined three patient groups based on the WHO 2017 morphological classification and the presence of *TERT* promoter mutations. During the median follow-up of 14.8 years, the HRs of CSS significantly increased in groups 2 and 3, whereas the HRs were not significantly different between the MI-FTC and EA-FTC WHO 2017 groups. Furthermore, the PVE and C-index of CSS were higher in the alternative groups than in the WHO-2017 groups, which suggested that the alternative group had better predictability for CSS in patients with FTC. Illustrations of each of three WHO 2017 classification with or without *TERT* promoter mutation were shown in Fig. 6.

*TERT* promoter mutations are associated with enhanced telomere maintenance, and cancer cells can be immortalized by maintaining the telomere length^29^. Previous studies have shown the association that *TERT* promoter mutations are associated with tumor aggressiveness and patients survival in DTC^16–18,21,30,31^. The *BRAF*^V600E^ mutation is considered to be a poor prognostic molecular marker of PTC^20^. Furthermore, only one case of a patient with FTC carrying a *BRAF*^K601E^ mutation has been reported^8^. Despite these differences, previous studies evaluated FTC to be considered as DTC, and most of the results from thyroid cancer were predominantly PTC. Considering that FTC showed poorer survival outcome than PTC, and that both have different clinical and molecular characteristics, independent risk-group stratification is needed.

Although the WHO 2017 classification of FTC is well-accepted and has improved prognostication by incorporating the importance of vascular invasion, its prognostic implications in CSS are still controversial. For the EA-FTC patients in this study, CSS differed significantly depending on the *TERT* promoter mutations (log-rank *P* < 0.001). Thus, we harmonized the WHO 2017 classification and *TERT* promoter mutations, which are promising molecular prognostic markers, and re-classified the thyroid carcinomas into three alternative groups. Considering that the WI-FTC presented with the most aggressive histology, it was interesting to see that the WI-FTC with WT-*TERT* patients (group 2) showed better clinical outcome than the EA-FTC with M-*TERT* patients (group 3). Furthermore, the proportion of *TERT* promoter mutations varied between the MI-, EA-, and WI-FTC groups (10.3, 20.8, and 35.7%, respectively). Therefore, we believe that the presence of vascular invasion may reflect the aggressiveness of *TERT* promoter mutations.

Although the alternative groups were proposed to optimize CSS prediction, they also assisted in the prediction of structural recurrence. O’Neil et al. reported that the 10-year DFS of MI-, EA-, and WI-FTC patients was 97, 81, and 46%, respectively^32^. Likewise, in the present study, 10-year DFS was 95.2, 85.6, and 42.9% in the MI-, EA-, and WI-FTC groups, respectively. However, the PVEs for the WHO 2017 groups were lower than those for the alternative groups. The PVE for DFS was 27.1 in the alternative groups, and the discrimination of HRs was increased in the alternative groups. Notably, there was no distinction between the MI- and EA-FTC WHO 2017 groups (*P* = 0.587), whereas significant distinctions were observed between the alternative groups 1 and 2 (*P* = 0.037). Given the favorable outcomes in the majority of FTC patients, identifying patients with a poor expected prognosis is a priority in clinical practice.

This study has several limitations. First, this study is retrospective in nature and was conducted in a single tertiary referral center. Thus, it is prone to selection bias. Second, this study was conducted with a relatively small number of patients, because the prevalence of FTC is relatively low in iodine-sufficient areas of South Korea. Therefore, external validation is encouraged using large population data sets. However, there were no previous reports about long-term follow-up data on *TERT* promoter mutations as prognostic marker in patients with FTC.

In conclusion, the alternative groups show clinical implications for CSS in patients with FTC. Currently, none of the mortality risk systems incorporate molecular markers as prognostic factors in thyroid carcinoma, even though new robust molecular classifications have been proposed for other cancers^33,34^. This study demonstrated that promising new molecular prognostic markers can be incorporated into the WHO 2017 classification system to better predict CSS as well as to increase DFS predictability. The results obtained in the present study suggest that *TERT* promoter mutation tests should be performed in patients with histologically confirmed EA- or WI-FTC.

## Supplementary information

Supplementary tables

## Data Availability

The datasets generated and/or analyzed during the current study are not publicly available but are available from the corresponding author on reasonable request.
