# Supplementary material for: Molecular classification of follicular thyroid carcinoma based on TERT promoter mutations
Source: Mod Pathol. 2021 Sep 8;35(2):186–92. doi: 10.1038/s41379-021-00907-6 (PMC8786663; doi:10.1038/s41379-021-00907-6)
Supplement: Supplementary file 1 — Supplementary tables [file 41379_2021_907_MOESM1_ESM.pdf]

Supplementary table 1. Hazard ratios of cancer-specific survival according to the WHO 2017 classification and the presence of *TERT* promoter mutations.

Staging	No of patients (*n*)	No of death (*n*)	CSS 5-year (%)	CSS 10-year (%)	CSS 15-year (%)	Hazard ratio (95% CI)	*P*-value
MI-FTC with WT-*TERT*	35	1	100	94.7	94.7	Reference	
EA-FTC with WT-*TERT*	19	1	100	93.3	93.3	1.21 (0.08-19.34)	0.893
WI-FTC with WT-*TERT*	9	3	88.9	77.8	66.7	7.13 (0.74-68.59)	0.089
MI-FTC with M-*TERT*	4	0	100	100	100	0.0	0.990
EA-FTC with M-*TERT*	5	3	33.3	0.0	0.0	59.09 (5.72-610.68)	0.001
WI-FTC with M-*TERT*	5	3	80.0	40.0	40.0	23.26 (2.33-231.78)	0.007

WHO World Health Organization, *MI-FTC* minimally invasive follicular thyroid carcinoma, *EA-FTC* encapsulated angioinvasive follicular thyroid carcinoma, *WI-FTC* widely invasive follicular thyroid carcinoma, *WT* wild-type, M mutant, *CSS* cancer-specific survival, *CI* confidential interval.

Supplementary table 2. Hazard ratios of disease-free survival according to the WHO 2017 classification and the presence of *TERT* promoter mutations.

Staging	No of patients (*n*)	No of recur (*n*)	DFS 5-year (%)	DFS 10-year (%)	DFS 15-year (%)	Hazard ratio (95% CI)	*P*-value
MI-FTC with WT-*TERT*	35	2	100	94.4	88.5	Reference	
EA-FTC with WT-*TERT*	19	1	100	93.3	93.3	0.68 (0.06-7.54)	0.753
WI-FTC with WT-*TERT*	9	3	66.7	66.7	66.7	5.19 (0.86-31.19)	0.072
MI-FTC with M-*TERT*	4	1	100.0	100.0	100.0	3.11 (0.28-34.70)	0.357
EA-FTC with M-*TERT*	5	2	0	0	0	22.32 (2.63-189.81)	0.004
WI-FTC with M-*TERT*	5	5	0	0	0	57.79 (9.24-361.54)	<0.001

WHO World Health Organization, *MI-FTC* minimally invasive follicular thyroid carcinoma, *EA-FTC* encapsulated angioinvasive follicular thyroid carcinoma, *WI-FTC* widely invasive follicular thyroid carcinoma, *WT* wild-type, *M* mutant, *DFS* disease-free survival, *CI* confidential interval.

Supplementary table 3. Clinicopathological characteristic of 77 patients according to alternative grouping.

	Group 1 (*n* = 58)	Group 2 (*n* = 9)	Group 3 (*n* = 10)	*P* for trend
Sex (*n*, %)				
Female	44 (74.6)	6 (66.7)	9 (90.0)	0.499
Male	14 (24.1)	3 (33.3)	1 (10.0)	
Age, year (mean, SD)	39.2 (14.1)	44.3 (11.4)	58.4 (16.4)	0.002*
Size				
Mean, cm (mean, SD)	3.4 (1.5)	4.2 (3.4)	5.4 (2.8)	0.110*
4 cm or less	40 (69.0)	7 (77.8)	4 (40.0)	0.152
More than 4 cm	18 (31.0)	2 (22.2)	6 (60.0)	
Gross ETE				
Absent	58 (100.0)	9 (100.0)	7 (70.0)	<0.001
Present	0 (0.0)	0 (0.0)	3 (30.0)	
Distant metastasis				
Absent	58 (100.0)	7 (77.8)	4 (40.0)	<0.001
Present	0 (0.0)	2 (22.2)	6 (60.0)	
AJCC/TNM 8th stage				
Stage 1	56 (96.6)	7 (77.8)	2 (20.0)	
Stage 2	2 (3.4)	2 (22.2)	5 (50.0)	<0.001
Stage 3/4	0 (0.0)	0 (0.0)	3 (30.0)	
Surgical extent				
Total	28 (48.3)	8 (17.4)	10 (100.0)	<0.001
Subtotal or lobectomy	30 (51.7)	1 (11.1)	0 (0.0)	
Cumulative RAI dose				
Less than 100 mCi	30 (51.7)	1 (2.9)	3 (30.0)	0.060
100 mCi or more	28 (48.3)	8 (88.9)	7 (70.0)	

SD standard deviation, *ETE* extrathyroidal extension, *AJCC/TNM* American Joint Committee/tumor-node-metastasis, *RAI* radioactive iodine, * *P* for trend for continuous variables was analyzed using Jonckheere-Terpstra test.
